# Heavy Metal Contamination in Soil and Brown Rice and Human Health Risk Assessment near Three Mining Areas in Central China

**DOI:** 10.1155/2017/4124302

**Published:** 2017-05-15

**Authors:** Yu Fan, Tingping Zhu, Mengtong Li, Jieyi He, Ruixue Huang

**Affiliations:** Department of Occupational and Environmental Health, Xiangya School of Public Health, Central South University, Changsha 410078, China

## Abstract

**Background:**

Metal mining and waste discharge lead to regional heavy metal contamination and attract major concern because of the potential risk to local residents.

**Methods:**

This research was conducted to determine lead (Pb), cadmium (Cd), arsenic (As), manganese (Mn), and antimony (Sb) concentrations in soil and brown rice samples from three heavy metal mining areas in Hunan Province, central China, and to assess the potential health risks to local inhabitants.

**Results:**

Local soil contamination was observed, with mean concentrations of Cd, Pb, Sb, and As of 0.472, 193.133, 36.793, and 89.029 mg/kg, respectively. Mean concentrations of Cd, Pb, Sb, Mn, and As in brown rice were 0.103, 0.131, 5.175, 6.007, and 0.524 mg/kg, respectively. Daily intakes of Cd, As, Sb, Pb, and Mn through brown rice consumption were estimated to be 0.011, 0.0002, 0.004, 0.0001, and 0.0003 mg/(kg/day), respectively. The combined hazard index for the five heavy metals was 22.5917, and the total cancer risk was 0.1773. Cd contributed most significantly to cancer risk, accounting for approximately 99.77% of this risk.

**Conclusions:**

The results show that potential noncarcinogenic and carcinogenic health risks exist for local inhabitants and that regular monitoring of pollution to protect human health is urgently required.

## 1. Introduction

Heavy metals such as lead (Pb), cadmium (Cd), manganese (Mn), and metalloids such as arsenic (As) and antimony (Sb) are found naturally in the earth. However, with increasing human activities, especially mining and industrial processing, these metals have become a worldwide environmental problem [1, 2]. Heavy metal contamination of the environment, especially soil, has been one of the most challenging pollution problems because of the severe toxicity, wide distribution, persistence, and transferability to plants of these metals compared with other pollutions and can lead to various diseases [3–6]. Li investigated toxic heavy metal concentrations in soils around a smelter in southern China and found significant pollution of local soils by Pb, Cd, As, Sb, and mercury (Hg), with maximum concentrations of 2485, 75.4, 71.7, and 2.58 mg/kg, respectively [7]. Wu et al. found significant heavy metal contamination in farmland soil in Du'an County in China, with concentrations in 74.6% of soil samples exceeding grade II of the Chinese National Soil Environmental Quality Standard (GB 15618-1995). Cd concentrations were found to be 70.6% higher than permitted by the standard, whereas As, nickel, zinc (Zn), chromium, Sb, copper, and Pb concentrations were 70.6%, 42.9%, 34.9%, 19.8%, 19.6%, 2.94%, 1.59%, and 0.79% higher, respectively [8]. Wu et al. [9] investigated the area around an abandoned e-waste recycling site in Longtang, South China, and found Cd concentrations > 0.39 mg/kg in the surface soil, which exceeded guideline levels. A study from the Zhao research group indicated that 99% of paddy soil samples from Nanxun County had Cd levels exceeding the natural background value and that Cd accumulation in local soils was widespread and spatially variable [10]. Li found Pb concentrations exceeding the threshold for arid agricultural soils in China by 72.46% in some villages located in central Gansu Province, China [11]. Unlike Pb and Cd, which have been identified to their toxicity and are unfavorable to human health, Zn, Cu, and Cr play significant biological roles, where zinc is a part of the superoxide dismutase enzyme, who has a role as an antioxidant, as well as takes part in neurotransmitters, and plays a key role in immune function, sound growth, and development, copper is essential for collagen synthesis, and chromium is involved in glucose metabolism.

Although some heavy metals in soils are, to some extent, essential for plant and crop growth, others are highly toxic to humans. Soils with toxic heavy metal contamination lead to plant absorption and accumulation of higher concentrations of heavy metals, which ultimately pass into the human body via the food chain [12]. The main exposure pathway of heavy metal risk is through food consumption, exceeding those through air inhalation and skin absorption.

China is the largest producer and consumer of rice in the world, contributing about 30% of the total global rice output. As a cereal grain, rice is the most widely consumed staple food, supporting a large part of the world's human population, especially in Asia [13, 14]. However, the consumption of rice contaminated with toxic heavy metals has been reported to be linked closely to health impacts. Horiguchi [15] found that Cd exposure through the consumption of self-grown rice presented a high risk to local Japanese farmers and that female farmers over 70 years of age in particular had decreased renal tubular function. Itai-itai disease, related famously to environmental pollution, is caused by Cd poisoning caused by local mining activities in Japan [12]. Local rice was found to be polluted by Cd, and local people who acquired Cd poisoning through the food chain had spinal and leg pain with complications including coughing, anemia, and kidney failure, ultimately leading to death [16]. According to the National Environmental Quality Standard for Soil, there are three levels. The first level, also called natural background, states that Pb is under 35 mg/kg, Cd is under 0.20 mg/kg, Hg is under 0.15 mg/kg, As is under 15 mg/kg, and Cr is under 90 mg/kg. The second level states that Pb is under 250–400 mg/kg, Cd is under 0.30–1.00 mg/kg, Hg is under 0.30–1.00 mg/kg, As is under 20–30 mg/kg, and Cr is under 250–400 mg/kg. The third level states that Pb is under 500 mg/kg, Cd is unavailable, Hg is under 1.50 mg/kg, As is under 30 mg/kg, and Cr is under 400 mg/kg.

Hunan Province, located in central China, is rich in mineral resources and is known as the “home of nonferrous metals.” The province contains more than 140 mineral deposits, with an exploitation history of more than 2700 years [17]. The world's largest Sb mine is located in Lengshuijiang (LSJ) County, in a remote mountainous area of Hunan Province. The massive Shuikou Mountain Pb/Zn mine, opened in 1896, is well known in China. A large exploited pyrite deposit is located at Liuyang (LY) Qibao Mountain. Because large numbers of mining companies, smelters, and refineries operate in this province, industrial activities including smelting and slag disposal may lead to soil contamination by heavy metals [18]. Hunan Province also has a high level of rice production, and food safety issues due to heavy metal contamination have received attention since the discovery of “Cd rice.” The estimated average dietary intake of Cd via rice consumption is 179.9 mg/day/person, which far exceeds the allowable limit specified by the World Health Organization (WHO) [19]. The reported incidence of lung cancer in Hunan miners with moderate or severe bronchoepithelial hyperplasia found in sputum is 10.8%, which is much higher than that of other cases (1.4%) [20]. Another study found that long-term environmental exposure to Cd and Pb was associated with increased risks of all-cause and all-cancer mortality [21].

Some studies of toxic metals such as Cd, As, and Pb in rice and soil in Hunan Province have focused on single metals or sampling sites. However, the cancer risk (CR) and health risk factors from various mines, various heavy metals, and multiple media have not been assessed systematically. Thus, this study assessed the risk posed by concentrations of five toxic heavy metals (Pb, Cd, As, Mn, and Sb) near three main mines in Hunan Province. Detailed sampling was conducted, the levels of heavy metals in soil and brown rice from areas near the mines were determined, and the health risk to local residents was assessed according to potential carcinogenic and noncarcinogenic risk factors. This study provides information on toxic heavy metals in rice and soil around mining sites as a foundation for comparison with other regions in China and worldwide, which could be useful for heavy metal contamination control and risk management in similar areas around the world [22].

## 2. Materials and Methods

### 2.1. Study Area and Sampling

**Figure 1:**
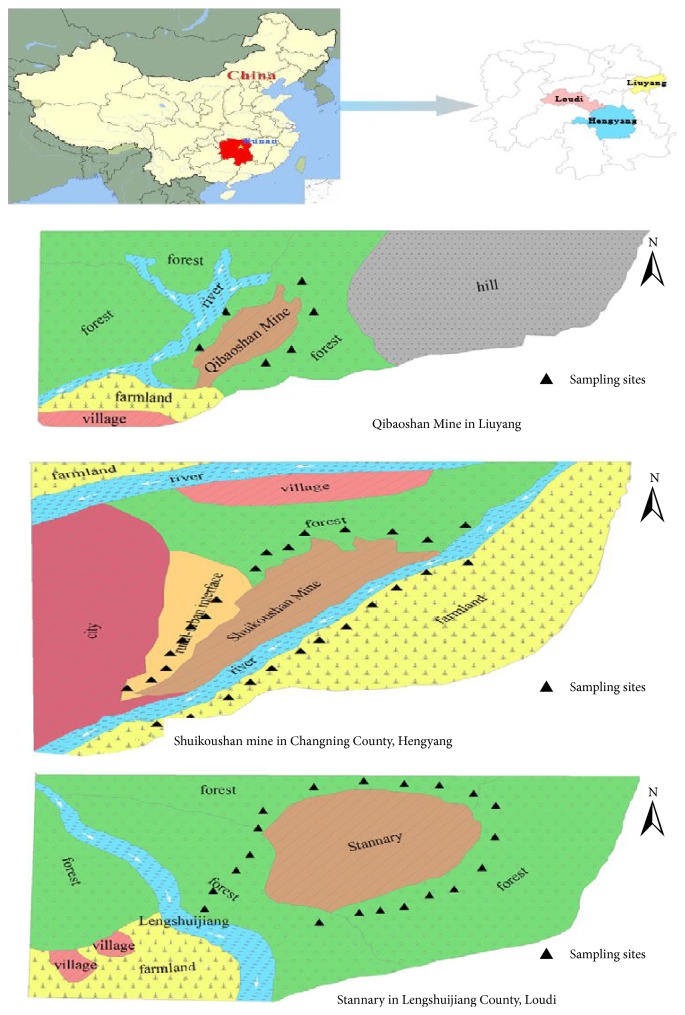
Map of three mines and sampling sites.

A total of 54 pairs of rice grain and soil samples were collected from LY (*n* = 6), Hengyang (HY; *n* = 28), and LSJ (*n* = 20) counties from August to October in 2013. Rice samples were harvested directly. LY is located in northeastern Hunan Province; it has an area of about 5007.75 km^2^, an estimated population of 1,407,104, and 27 towns and 6 townships. The geographical coordinates of LY are 27°51′–28°34′ N and 113°10′–114°15′ E (Figure 1). HY is located in southern Hunan Province, with the geographical coordinates of 26°45′36″ N and 112°38′06″ E. LSJ is located in western Hunan Province, and has an area of 436.28 km^2^. The geographical coordinates of LSJ are 27°41′10″ N and 111°26′10″ E (Figure 1). LSJ is rich in Sb and has the world's largest Sb mine (contributing >50% of the world's Sb production). Soil samples from the three mines were taken at a depth of 20 cm from the surface; each sample was a composite of 20 subsamples taken across a 1 × 1 m^2^ area [23]. All collected soil samples were placed into plastic bags and stored in cool, dry conditions, then transported to the laboratory for further assessment. Rice samples were collected from local inhabitants around the three mine areas. The soil and rice samples were 1 kg each.

### 2.2. Sample Preparation and Analysis

Soil samples were dried naturally in the laboratory at room temperature, then sieved using a 0.2 mm (100 mesh) nylon sieve. Rice grains were oven dried for 48 h, then milled using a rice-milling machine to obtain brown rice. The brown rice samples were sieved through a 60-mesh nylon sieve and stored in polyethylene flasks for digestion.

The concentrations of Pb, Cd, Mn, As, and Sb were analyzed. First, each sample was digested by adding 5–10 g sample to a 100 mL round-bottomed flask with 10 mL concentrated nitric acid and heating to 220°C. Hydrogen peroxide (1 mL) was added periodically until digestion was complete; therefore, a clear solution was obtained. Finally, each sample solution was diluted to 50 mL with distilled water in a volumetric flask. Cd and Pb concentrations in the solutions were analyzed by graphite furnace atomic absorption spectroscopy (TAS-990 Super, Beijing, China). Flame atomic absorption spectroscopy (TAS-990 Super) was used to determine Mn concentrations. Hydride atomic absorption spectrometry (TAS-990 Super) was used to analyze As and Sb concentrations [24, 25].

Quality assurance and quality control were important to ensure the reliability of measurements. In this study, all experimental reagents used were of analytical grade. Deionized water was used throughout the experimental procedure. Each soil and rice sample was analyzed in triplicate, and one standard sample was analyzed after every three experimental samples to ensure the accuracy of the results. Field blank and experimental blank samples were also analyzed to ensure the accuracy of data obtained. The accuracy of the soil sample analytical procedure was verified by the analysis of GSS-8, and the rice sample analytical procedure was verified by the analysis of GBW(E)080684. Recoveries were ±15% of actual values.

### 2.3. Transfer Factor

The transfer factor (TF) is an index describing the transferability of heavy metals from soils to the human body via the food chain [24]. It is represented using the ratio of a heavy metal concentration in a plant to the total metal concentration in the soil. The TF can be used to estimate potential human health risks posed by heavy metals. It is calculated as follows:

(1) $$\begin{array}{c} TF=\frac{Cr}{Cs}, \end{array}$$

where Cr is the heavy metal concentration in rice extracts and Cs is the heavy metal concentration in soil extracts.

### 2.4. Estimated Daily Intake of Heavy Metals through Brown Rice Consumption

The average daily intake [ADD, mg/(kg/day)] is a parameter used to quantify the oral exposure dosage during a specific period, expressed as a daily dose per unit body weight. The daily heavy metal intake dose depends on the heavy metal concentration and the amount of any respective food consumed. It is usually calculated using the following formula:

(2) $$\begin{array}{c} ADD=\frac{C\times IR\times EF\times ED}{BW\times AT}, \end{array}$$

where *C* is the mean heavy metal dose (mg/kg) and IR, ED, EF, BW, and AT represent the ingestion rate [22, 26–28], exposure duration, exposure frequency, reference body mass, and average time, respectively. The IR was estimated to be 0.425 kg, according to the average daily rice intake of adults in Hunan Province; the BW of 58.1 kg was taken from data in related studies [22, 29–31]; the ED was 365 days; the EF was 74 years (based on age); and the AT was 27,010 (the product of 365 days and an age of 74 years).

### 2.5. Human Health Risk Assessment

#### 2.5.1. Noncarcinogenic Risk

To analyze human noncarcinogenic risk for local inhabitants, the reference dose (RfD), which is the United States Environmental Protection Agency's (USEPA's) maximum acceptable oral dose for a toxic substance, was used. The hazard quotient (HQ), a ratio of the ADD to the RfD [31], represents the health risk of noncarcinogenic adverse effects due to exposure to toxicants:

(3) $$\begin{array}{c} HQ=\frac{ADD}{RfD}. \end{array}$$

Here, the RfD is an index of the estimated maximum permissible dose for humans through daily exposure. HQs < 1 can be assumed to reflect safety, whereas HQs > 1 are assumed to indicate potential noncarcinogenic effects [31]. However, to assess the potential risk of adverse health effects from a mixture of chemical elements in brown rice, the hazard index (HI), arising from the sum of HQs, was calculated:

(4) $$\begin{array}{c} HI=\sum HQ. \end{array}$$

HIs < 1 indicate that chronic risks are unlikely, whereas HIs > 1 indicate that noncancerous risks are likely to occur.

#### 2.5.2. Carcinogenic Risk

A cancer slope factor (SF) and the ADD were used to calculate the CR, as shown in (5). CR is estimated as the incremental probability of an individual developing cancer over a lifetime. For example, a CR of 10^−4^ indicates the probability that 1 in 10,000 individuals will develop cancer. The CR of local people caused by potential carcinogen exposure over a lifetime was calculated according to the following equation [32, 33]:

(5) $$\begin{array}{c} CR=ADD\times SF. \end{array}$$

In the real world, CR estimation should consider the effects of multiple carcinogenic elements. Thus, the sum of CRs from all carcinogens was summed and reported as total cancer risk (CRt):

(6) $$\begin{array}{c} CRt=\sum CR. \end{array}$$

According to the International Agency for Research on Cancer (IARC), Cd, As, and Sb are potentially carcinogenic substances, whereas Pb is classified as a class 2B potential carcinogenic substance and Mn is noncarcinogenic substance.  Table 1 shows the oral RfD and SF values for heavy metals in food.

## 3. Results

### 3.1. Heavy Metal Concentrations in Soil

The concentrations of five heavy metals in soils from around the three mines are shown in Table 2. Mean concentrations of Cd were greater than 0.3 mg/kg, which is the corresponding maximum allowable concentration (MAC), and mean values of As were more than three times higher than the corresponding MAC. Although concentrations of Pb were below the MAC, they were higher than the corresponding natural background values. Mn concentrations were lower than the background value, suggesting that soils around these three mining areas contain no Mn pollution.

Significant variability in Cd, As, and Pb soil concentrations near the three mine areas was observed. The HY mine area had the maximum mean Cd concentration of 0.865 mg/kg, approximately 17 times the concentrations found at LY and LSJ. The mean level of As in HY was 142.714 mg/kg, which exceeded the values from LY and LSJ by about five times. LSJ had the highest Sb level among the three mine areas, whereas HY had the highest level of Pb. These results demonstrate that the extent of contamination differed substantially among the three mine areas.

### 3.2. Heavy Metal Concentrations in Brown Rice

Concentrations of Cd, Pb, Sb, Mn, and As in brown rice near the three mines are shown in Table 3. Among these study areas, the Mn level in rice was highest in the LY region, whereas the Sb level in rice was highest in HY. The mean concentrations of Mn and Sb in brown rice were 5.934 and 7.901 mg/kg, respectively.

### 3.3. Transfer Factors

**Figure 2:**
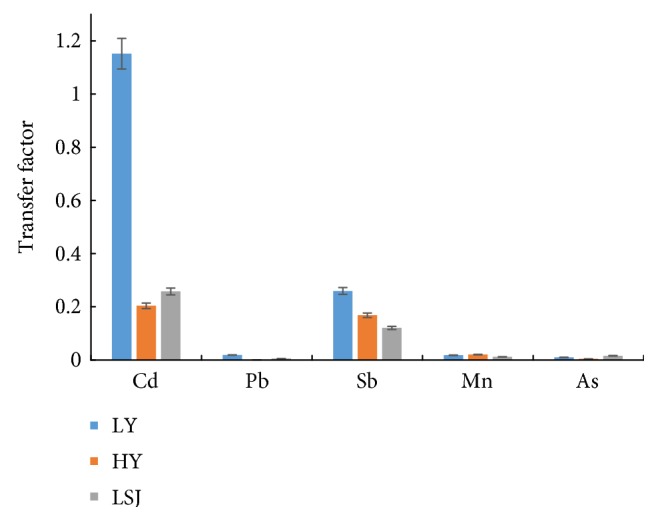
Transfer factor of Cd, Pb, Sb, Mn, and As in soil near three mines.

Figure 2 shows the TFs of Cd, Pb, Sb, Mn, and As near the three mines. Different heavy metals had different TFs, with a decreasing trend in the order of Cd > Sb > Mn > Pb > As. Cd had the highest TF of 1.151, followed by Sb (TF = 0.259).

### 3.4. Health Risks to Residents from Heavy Metal Exposure in Brown Rice

The ADD values for Cd, Pb, Sb, Mn, and As from brown rice consumption were calculated to be 0.75, 0.96, 37.85, 43.94, and 3.83 mg/kg, respectively. The ADDs of Mn, Pb, and Sb were thus low, and adult daily intake of these contaminants from rice was not significant. However, the ADD for Cd was much higher than those for the other four heavy metals, which suggested a large adult daily intake of Cd from rice.

The HQs for Cd, Pb, Sb, Mn, and As through brown rice consumption for local residents were 11.798, 0.0484, 10.0158, 0.0025, and 0.7264, respectively. The HQs of Cd and Sb exceeded 1, suggesting that these metals pose potential noncarcinogenic risks for local people. The other elements had no obvious individual risk, but the combined HI value for all five elements was 22.5917, implying a high noncarcinogenic health risk and chronic toxicity due to combined exposure to these heavy metals in brown rice.

CR values for Cd and As were 0.1769 and 0.0003, respectively. The Cd value exceeded 10^−4^, and the CRt was 0.1773, indicating a high potential carcinogenic risk from brown rice consumption. PLI, pollution load index, has been calculated in the study based on the below equation and in the soil; the outcome is 6.028 whereas in rice is 2.53.

$PLI=\sqrt[{n}]{{CF}_{1}\times {CF}_{2}\times \cdots \times {CF}_{n}}\;$ (*n*, heavy metal types).

## 4. Discussion

Hunan Province, which has a large nonferrous metal industry, abounds in minerals, including Pb, Zn, Sb, coal, Hg, and gold ores, and has more than 2000 years of metal mining and smelting history [35]. Mining in Hunan has contributed significantly to the growth and development of the economy and society, but because mining and industrial activities result in the discharge of large amounts of heavy metal wastewater, gases, and residues, environmental contamination issues have arisen for the government and for local residents. Li [36] reported that concentrations of Hg and Pb in soils in Hg deposit areas were 1.315 and 3.1 times higher, respectively, than those of the average background of soils in China and that concentrations were 14.8 and 16.1 times higher, respectively, in soils in Pb/Zn deposit areas. Du found that the total Cd content in the soils of the county ranges from 0.13 to 6.02 mg/kg and that 57.5% of soils had concentrations exceeding the allowable limit specified by the China Soil Environmental Quality Standards. He et al. [37] reported that the mean soil concentrations of Cd, As, and Pb in Hunan Province were 0.299, 15.9, and 40.5 mg/kg, respectively, which all exceed the Chinese Environmental Quality Standard for Soil, grade II (GB 15618-1995), as well as the corresponding background values in Hunan. The results of our study are consistent with those of previous studies showing Cd, Pb, As, and Sb heavy metal contamination exceeding the background values in Hunan Province. However, some soil heavy metal concentrations showed great variation across the three mining areas; this variation may be due to local geology and the influence of human mining activities. In HY, abundant reserves of minerals with uncontrolled Pb, Zn, and As mining led to the release of Pb, As, and other heavy metals and contaminated soils around mines, with the degree of contamination possibly aggravated by geographical features such as large rain fall, typically steep hillsides, and severe soil erosion [38]. These results were consistent with those of related studies [39, 40]. The mean Sb concentration in LSJ was higher than those in the other two mining areas and greatly exceeded the maximum permissible soil concentration for Sb recommended by the WHO of 36 mg/kg. This result indicates that contamination of soil by Sb in LSJ is serious. Average concentrations of Mn were lower than the corresponding background values, indicating slight pollution in local soils compared with the other four heavy metals.

Rice is a major food crop for 3 billion people, especially those in Asian countries. Rice has been implicated as a major route for heavy metal exposure, especially in mining areas. The result of rice was higher in nonmining areas. Ma et al. [41] collected rice samples from 40 counties and found that the mean total As concentration was 0.129 mg/kg; hence, one can conclude that the consumption of locally grown rice is a major source of As exposure for local residents. Cd and Pb levels in rice were lower than the MACs; these results may have been influenced by the choice of sampling sites or affected by other areas with severe heavy metal pollution. Further studies should be undertaken with more samples. Although our study showed that Cd and Pb levels in rice were low, many other studies have shown high Cd and Pb concentrations in rice [42, 43]. A “Cd rice” incident occurred in 2014 in Youxian County, Hunan Province, and caused significant social panic. Compared with other reports studied in the same province which have had evidence that the concentrations of heavy metal are greatly high in the food, our results are similar to those of the previous reports. All the soil samples' TFs are beyond 1, which means there exists a significant heavy metal pollution in soils. 66.6% Tfs of rice samples are beyond 1, which indicates that different heavy metals have different transferability.

The USEPA has provided an oral RfD of 0.4 *μ*g/kg body weight/day for Sb. According to the WHO, rice contributes 33% of the total daily intake of Sb, which is higher than from other exposure routes. The Sb concentrations in rice near the Shuikoushan mine were 3.507–10.320 mg/kg, whereas Qu et al. [44] found that the Sb level in local rice was 0.47 mg/kg. These results show greater Sb contamination near the Shuikoushan mine compared with the other Sb mine, where Sb concentrations were in the range of 0.160 to 0.930 mg/kg. These results could help residents around LSJ and policy makers in taking measures to alleviate health risks in mining areas.

The TFs of heavy metals are usually used as indicators of heavy metals in soils that quantify the bioavailability of heavy metals to agricultural products, such as brown rice. The results of this study showed greater mobility of Cd and Sb from soil to rice, followed by Mn, Pb, and As. The TF indicates a key process for human exposure to toxic heavy metals through the food chain [45–47]. Our results indicated that Cd and Sb contamination in soil poses a major human health risk.

The HQ and CR are often used to represent potential human health risks. The HI is used to indicate potential noncancerous risks. In this study, the HI for brown rice consumption of the five heavy metals was high (22.5917), indicating that uptake through local brown rice consumption may present a significant noncarcinogenic risk. This result is similar to that reported by Qu, who found that heavy metal pollution may pose significant potential health risks to local residents, especially in the village closest to the mine. The HI can likely be explained mainly by Cd and Sb contamination, as their HQs accounted for 52.22% and 44.26%, respectively, of this value. These results indicate that Cd and Sb pose high noncarcinogenic risks to human health. Therefore, perennial intake of contaminated brown rice by local inhabitants near mines is likely to pose a relatively high risk to health.

The IARC has classified Cd and Cd compounds as carcinogenic to humans. As-containing compounds, such as potassium arsenite, are highly toxic and carcinogenic to humans. In this study, the CRt for the five heavy metals was 0.1773, more than 400 times the limit. This finding reflects a potentially high carcinogenic risk to local inhabitants through consumption of local brown rice. Notably, Cd contributed approximately 99.77% to the CR. Wongsasuluk et al. [48] studied mortality in a population living around a multimetal sulfide mine in Guangdong Province, and found that long-term environmental exposure to Cd led to an increased risk of mortality from all cancers. Further control measures should be implemented to decrease Cd concentrations in soils and rice, thereby reducing the CR and providing a safe source of food to people living around mining areas. Hunan Province has a long mining history and named as “heavy metal town” for over thousand years. Long-term mining activities contribute to change the ingredients of the soils, and the heacy metals can be concentrated into the rice through soil and water or air. In this study, the CRt for the five heavy metals was 0.1773, more than 400 times the limit, which support the evidence that long-term mining has a harmful effect for local residents.

Other intake pathways for heavy metals include those through vegetables, fish, poultry, and water [49]. Therefore, further studies on other intake pathways should be conducted in order to evaluate the risk assessment comprehensively. Based on the above discussion, measures must be taken to ensure the safety of local food and reduce the risks from other exposure pathways.

Considering the limited number of soil and rice samples analyzed in this study, further studies are needed to enhance our understanding of human dietary exposure to heavy metals in these mine areas.

## 5. Conclusions

This study evaluated the human health risk due to concentrations of five heavy metals in soils and rice near three mining areas in Hunan Province. The results are critically serious. Although some strategies, such as dust control, occupational health measures, and monitoring of occupational hazards, are conducted to limit environmental heavy metal contamination around these three mining areas, those strategies need to be strengthened and broadened to reduce the toxic effects of Sb and Cd exposure for workers and local residents. Thus, we strongly recommend that effective measures, including remediation of contaminated soil to ensure edible crops, should be taken in communities near the three mining areas. Meanwhile, regular monitoring of heavy metals in soils and rice in these areas is strongly recommended.

## Figures and Tables

**Table 1:** Reference doses (RfD) and slope factors (SF) of five heavy metals.

Elements	Classification by IARC^a^	RfD (mg/(kg/day))	Source	SF (mg/(kg/day)^−1^)	Source
Cd	1	0.001	IRIS^b^	15	CALEPA^c^
As	1	0.003	IRIS	1.5	IRIS
Pb	2B	0.0036	WHO^d^	**—**	
Mn	—	0.14	IRIS	**—**	
Sb	2B	0.004	IRIS	**—**	

*Note*. ^a^The International Agency for Research on Cancer defines human carcinogens as class 1 and possible human carcinogens as class 2B. ^b^Integrated Risk Information System, USEPA. ^c^California Environmental Protection Agency, US. ^d^World Health Organization.

**Table 2:** Concentrations (mean ± standard deviation, mg/kg) of Cd, As, Sb, Pb, and Mn in soil near three mine areas in Hunan Province.

		Cd	As	Sb	Pb	Mn
LY (*n* = 6)	Mean ± SD	0.050 ± 0.001	26.495 ± 3 .2326	16.362 ± 1.458	30.337 ± 0.845	332.604 ± 3.662
	Range	0.006–0.107	17.690–34.102	10.110–22.300	17.345–41.450	241.030–401.260

HY (*n* = 28)	Mean ± SD	0.865 ± 0.052	142.714 ± 2.210	20.393 ± 1.577	356.161 ± 1.905	420.316 ± 8.631
	Range	0.345–1.451	26.870–562.340	9.620–36.400	124.360–756.200	215.630–666.310

LSJ (*n* = 20)	Mean ± SD	0.049 ± 0.001	32.628 ± 1.242	65.737 ± 5.340	13.732 ± 1.616	229.395 ± 7.921
	Range	0.010–0.130	12.540–70.230	37.100–111.240	0.555–33.405	102.650–412.860

Total	Mean ± SD	0.472 ± 0.194	89.029 ± 1.461	36.739 ± 7.223	193.133 ± 3.855	339.996 ± 15.924
	Range	0.006–1.451	12.540–562.340	9.620–111.240	0.555–756.200	102.650–666.310

Background value^a^		0.098	14		27	459
MAC^b^		0.3	25	36^d^	300	NV^c^

*Note*. ^a^Soil background value in Hunan Province and study methods (Pan et al., 1998). ^b^Maximum allowable concentration of heavy metals in soil, recommended by the Chinese Environmental Quality Standard for Soil, grade II (GB 15618-1995). ^c^No criterion value set [34]. ^d^Recommended in soils by the WHO (Chang et al., 2002).

**Table 3:** Concentrations of Cd, Pb, Sb, Mn, and As in brown rice near three mines.

		Cd	Pb	Sb	Mn	As
LY (*n* = 6)	Mean ± SD	0.058 ± 0.001	0.564 ± 0.089	4.245 ± 1.200	5.934 ± 1.042	0.261 ± 0.002
	Range	0.006–0.173	0.058–1.074	3.210–6.340	4.32–7.569	0.211–0.342

HY (*n* = 28)	Mean ± SD	0.176 ± 0.002	0.081 ± 0.001	7.901 ± 2.921	2.652 ± 0.464	0.586 ± 0.032
	Range	0.119–0.241	0.040–0.107	3.507–10.320	1.765–4.023	0.235–0.986

LSJ (*n* = 20)	Mean ± SD	0.013 ± 0.001	0.069 ± 0.003	5.175 ± 1.325	2.652 ± 0.464	0.517 ± 0.017
	Range	0.007–0.016	0.044–0.287	1.987–10.320	1.765–4.023	0.235–0.075

Total	Mean ± SD	0.103 ± 0.007	0.131 ± 0.034	5.175 ± 1.325	6.007 ± 1.552	0.524 ± 0.033
	Range	0.006–0.241	0.040–1.074	1.987–10.320	1.765–10.040	0.211–0.986
	MAC^a^	0.2^b^	0.2^b^	NV^c^	NV	0.5^b^

*Note*. ^a^Maximum allowable concentration of heavy metals in rice, recommended by Maximum Levels of Contaminants in Foods (GB 2762 2012). ^b^Zhao et al. [10]. ^c^Not available.
